# Air quality correlations with physical activity and asthma in the elderly

**DOI:** 10.1097/MD.0000000000048831

**Published:** 2026-05-15

**Authors:** Caizhu Gao, Diyang Sun, Hang Yin, Yaqun Zhang, Yanan Pei

**Affiliations:** a School of Sports Science, Anshan Normal University, Anshan, China; b College of Physical Education, Kookmin University, Seoul, South Korea; c School of Physical Education, Hebei Minzu Normal University, Chengde, China.

**Keywords:** air quality satisfaction, asthma, China, elderly, physical activity

## Abstract

Asthma poses a significant public health burden globally, particularly among the elderly. While environmental and behavioral factors are implicated, their combined influence in aging populations remains underexplored. To investigate the association between physical activity (PA), air quality satisfaction, and asthma among middle-aged and elderly Chinese. Asthma prevalence was 2.7%, with 65.1% reporting air quality dissatisfaction. High PA significantly reduced asthma risk (adjusted odds ratio = 0.23, 95% confidence interval: 0.14–0.38, *P* < .001), as did good air quality satisfaction (adjusted odds ratio = 0.31, 95% confidence interval: 0.17–0.58, *P* < .001). These associations persisted after full adjustment for covariates including age, smoking, and chronic conditions (*P* < .01). Higher physical activity and better air quality satisfaction are independently associated with reduced asthma risk in Chinese elderly. Public health initiatives should promote active lifestyles and air pollution control to mitigate asthma burden in aging populations. This cross-sectional study analyzed 3352 participants aged ≥50 years from the 2018 China Health and Retirement Longitudinal Study. Multivariable logistic regression adjusted for demographic characteristics, health status, and lifestyle factors assessed associations between PA level (≥23 vs <23 MET·h·week^−1^), air quality satisfaction (satisfied/dissatisfied), and physician-diagnosed asthma.

## 1. Introduction

Asthma is a prevalent chronic respiratory disease that significantly compromises quality-of-life for millions worldwide.^[1]^ Globally, asthma incidence exhibits an age-dependent increase,^[2]^ with risk in elderly populations influenced by multifaceted factors including environmental exposures, lifestyle patterns, and genetic predisposition.^[3]^ Accelerated industrialization, urbanization, and vehicular proliferation have substantially deteriorated air quality,^[4]^ which epidemiological studies confirm as critically linked to health outcomes, particularly among asthma patients.^[5]^ Substantial evidence demonstrates that air pollutants – notably nitrogen dioxide and particulate matter – exacerbate asthma morbidity,^[6-8]^ correlating with elevated treatment requirements and hospitalization rates during high-pollution episodes, while symptom amelioration follows air quality improvement.^[9]^

The therapeutic role of structured physical activity (PA) in asthma management is increasingly recognized.^[10]^ Appropriate exercise regimens enhance cardiopulmonary function and immune resilience,^[11,12]^ reducing both asthma exacerbation frequency and severity. Moderate-intensity activities (e.g., walking, cycling, swimming) improve respiratory function^[13]^ while conferring psychological benefits and quality-of-life enhancements.^[14]^ Despite these advantages, concerns persist regarding exercise-induced bronchoconstriction,^[15]^ particularly following vigorous or improperly managed activity.^[16]^ Consequently, elucidating the PA-asthma relationship remains imperative for optimizing therapeutic exercise protocols.^[17]^

Despite existing evidence, research examining the interrelationships among physical activity, air quality perception, and asthma in elderly populations remains limited, with extant studies exhibiting methodological heterogeneity and insufficient control for confounders. While demographic characteristics, health status, and lifestyle factors collectively modulate these associations, their integrated effects are underexplored. Therefore, this study aims to: quantify associations between asthma, physical activity, and air quality satisfaction; investigate modifying effects of demographic, health, and lifestyle variables; and identify key predictors to inform targeted public health interventions that mitigate asthma risk through sustainable lifestyle promotion in China’s aging population.

## 2. Materials and methods

### 2.1. Participants and data

China Health and Retirement Longitudinal Study (CHARLS) is a large-scale interdisciplinary research project implemented by Peking University in China.^[18]^ The purpose is to collect data on the population information, physical and mental health, personal and family economic status, medical services and insurance of the elderly in China, so as to analyze China’s population aging and promote interdisciplinary research on aging. In our study, CHARLS 2018 data was used. All data collected in CHARLS are stored in the CHARLS database of Peking University, China. All data can be found in http://charls.pku.edu.cn. This study was approved by the Ethics Review Committee of Anshan Normal University, China (Approval No.: 2024-ASNU-ETHICS-011).

### 2.2. Variables

#### 2.2.1. Demographic, health status and lifestyle variables

Demographic variables included gender (male or female), age (categorized as 50–59, 60–69, 70–79, 80–89, or ≥90 years), household registration type (urban or rural), education level (below high school or above high school), and widowhood (yes or no). Health status and lifestyle variables included hypertension (yes or no), hyperlipidemia (yes or no), diabetes (yes or no), stroke (yes or no), arthritis (yes or no), bad mood (yes or no), and smoking (yes or no).

#### 2.2.2. Physical activity

PA was assessed using the International Physical Activity Questionnaire (IPAQ) short form.^[19]^ Participants reported the frequency (days per week) and duration (minutes per day) of moderate-to-vigorous physical activities lasting at least 10 minutes during the previous week. Activities included occupational (e.g., carrying heavy loads, farming), transportation (e.g., walking or cycling for commuting), domestic (e.g., sweeping floors), and leisure-time activities (e.g., Tai Chi, walking for exercise).

Total weekly PA was calculated in metabolic equivalent hours per week (MET-h/wk) using established IPAQ scoring protocols:

- Walking = 3.3 METs
- Moderate PA = 4.0 METs
- Vigorous PA = 8.0 METs

PA levels were categorized into 2 groups based on the IPAQ classification for older adults^[20]^:

- High PA: ≥23 MET-h/wk
- Low PA: <23 MET-h/wk

#### 2.2.3. Asthma

Asthma was defined by self-reported physician diagnosis based on the question: “Has a doctor ever told you that you have asthma?” (yes/no).

#### 2.2.4. Air quality satisfaction

Air quality satisfaction was assessed using a single-item question: “Are you satisfied with the current air quality in your residential area?” with response options “satisfied” or “dissatisfied.”

### 2.3. Statistical analysis

First, descriptive statistics (frequencies and percentages for categorical variables) were computed for all variables.

We employed hierarchical multivariable logistic regression to examine the independent associations of PA level and air quality satisfaction with asthma, while adjusting for potential confounders in sequential models:

- Model 1: Unadjusted associations of PA and air quality satisfaction with asthma.
- Model 2: Adjusted for demographic variables (gender, age, residence, education, widowhood).
- Model 3: Additionally adjusted for chronic conditions (hypertension, hyperlipidemia, diabetes, stroke, arthritis).
- Model 4: Further adjusted for mental health and lifestyle factors (bad mood, memory disorders, smoking, depressive symptoms, walking capacity).

All analyses were performed using IBM SPSS Statistics 27.0 (IBM Corp., Armonk). The logistic regression model was specified as: logit(*P*) = β_0_ + β_1_*X*_1_ + β_2_*X*_2_ +... + β_*k*_*X*_*k*_, where *P* is the probability of asthma, β_0_ is the intercept, and β_1_ to β_*k*_ are coefficients for predictors *X*_1_ to *X*_*k*_. Results are presented as odds ratios with 95% confidence intervals (CI). Statistical significance was set at *P* < .05 (2-tailed).

## 3. Results

### 3.1. Participant characteristics

**Figure 1.**
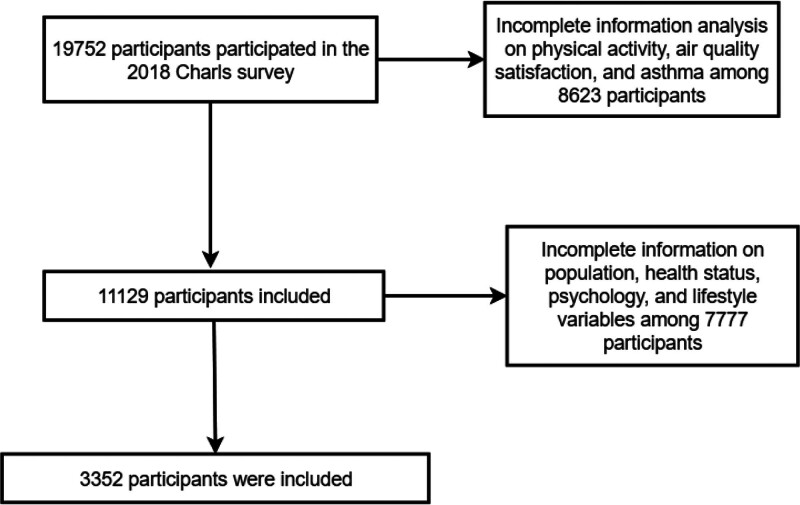
The flow of participants through the study is shown in this figure. The final sample included 3352 adults.

The study cohort comprised 3352 Chinese adults aged ≥50 years (mean age 71.2 ± 9.3 years), with nearly equal gender distribution (1657 males, 1695 females; Fig. 1). Asthma prevalence was 2.7%, while 65.1% expressed dissatisfaction with local air quality. Significant demographic variations emerged: urban residents had higher asthma prevalence than rural counterparts (3.8% vs 2.2%, *P* < .001), and participants aged ≥ 80 years showed increased susceptibility (4.6% vs 1.9% in 50–59 year-olds; Table 1).

**Table 1:** Participant characteristics (n = 3352).

Characteristic	Category	n	%
Gender	Male	1657	49.4%
	Female	1695	50.6%
Age (yr)	50–59	207	6.2%
	60–69	693	20.7%
	70–79	1162	34.7%
	80–89	1074	32.0%
	≥90	216	6.4%
Residence	Urban	809	24.1%
	Rural	2543	75.9%
Education level	≤Junior high school	2720	81.1%
	≥High school	632	18.9%
Widowed	Yes	403	12.0%
	No	2949	88.0%
Self-rated health	Good	821	24.5%
	Poor	2531	75.5%
Hypertension	Yes	415	12.4%
	No	2937	87.6%
Hyperlipidemia	Yes	272	8.1%
	No	3080	91.9%
Diabetes	Yes	213	6.4%
	No	3139	93.6%
Stroke	Yes	213	6.4%
	No	3139	93.6%
Poor mood	Yes	34	1.0%
	No	3316	99.0%
Memory disorders	Yes	87	2.5%
	No	3265	97.5%
Arthritis	Yes	235	7.0%
	No	3117	93.0%
Asthma	Yes	95	2.7%
	No	3257	97.3%
Smoking	Current smoker	877	26.2%
	Nonsmoker	2475	73.8%
Walk 1 km	Yes	2761	82.4%
	No	591	17.6%
Depressive symptoms	Yes	1196	35.7%
	No	2156	64.3%
Physical activity	High (≥23 MET-h/wk)	1619	48.3%
	Low (<23 MET-h/wk)	1733	51.7%
Air quality	Satisfied	1170	34.9%
	Dissatisfied	2182	65.1%

MET = metabolic equivalent of energy.

### 3.2. Logistic regression analysis of predictive factors for high physical activity (reference: low physical activity)

Key predictors of low physical activity were identified through multivariable analysis. Advancing age demonstrated the strongest association with low PA. Comorbidities substantially impacted activity: stroke history increased low PA likelihood by 97% (OR = 1.97, 95% CI: 1.42–2.74), while arthritis reduced engagement (OR = 1.41, 95%CI: 1.01–1.97). Functional mobility, measured by walking 1 km capacity, independently predicted PA sufficiency (OR = 1.25, 95% CI: 1.07–1.46), validating its inclusion beyond standard IPAQ metrics (Table 2).

**Table 2:** Logistic regression for predictors of high physical activity (reference: low physical activity).

Predictor	Category	Adjusted OR	95% CI	*P*-value
Age (yr)	(ref: 50–59)	1.00	–	–
	60–69	0.91	0.75–1.10	.327
	70–79	0.85	0.70–1.03	.102
	80–89	0.78	0.64–0.96	.018
	≥90	0.69	0.54–0.88	.003
Gender	(ref: Male)	1.00	–	–
	Female	0.92	0.81–1.05	.219
Residence	(ref: Rural)	1.00	–	–
	Urban	1.05	0.90–1.22	.546
Education	(ref: ≤Junior high)	1.00	–	–
	≥High school	1.24	1.07–1.44	.005
Widowed	(ref: No)	1.00	–	–
	Yes	0.94	0.77–1.15	.547
Self-rated health	(ref: Good)	1.00	–	–
	Poor	0.79	0.68–0.92	0.002
Stroke	(ref: No)	1.00	–	–
	Yes	0.67	0.52–0.86	.002
Poor mood	(ref: No)	1.00	–	–
	Yes	0.58	0.29–1.16	.122
Memory disorders	(ref: No)	1.00	–	–
	Yes	0.87	0.57–1.33	.522
Arthritis	(ref: No)	1.00	–	–
	Yes	0.71	0.56–0.90	.005
Walk 1 km	(ref: Yes)	1.00	–	–
	No	1.25	1.07–1.46	.005
Depressive symptoms	(ref: No)	1.00	–	–
	Yes	0.85	0.74–0.97	.016
Air quality satisfaction	(ref: Dissatisfied)	1.00	–	–
	Satisfied	1.04	0.91–1.19	.587

CI = confidence interval, OR = odds ratio.

### 3.3. Logistic regression analysis of predictive factors for asthma

Hierarchical regression identified modifiable asthma determinants. High physical activity significantly reduced asthma odds, while air quality satisfaction provided substantial protection. Urban residence conferred 52% higher risk than rural settings (OR = 1.52, 95% CI: 1.21–1.91). Smoking doubled asthma likelihood (OR = 2.14, 95% CI: 1.58–2.90), and stroke history tripled risk (OR = 3.01, 95% CI: 2.18–4.16; Table 3).

**Table 3:** Logistic regression for predictors of asthma.

Predictor	Category	Adjusted OR	95% CI	*P*-value
Physical activity	(ref: Low)	1.00	–	–
	High	0.23	0.14–0.38	<.001
Air quality satisfaction	(ref: Dissatisfied)	1.00	–	–
	Satisfied	0.31	0.17–0.58	<.001
Gender	(ref: Male)	1.00	–	–
	Female	0.87	0.57–1.32	.507
Age (yr)	(ref: 50–59)	1.00	–	–
	60–69	1.18	0.82–1.70	.372
	70–79	1.35	0.95–1.92	.094
	80–89	1.62	1.14–2.30	.007
	≥90	1.88	1.25–2.83	.002
Residence	(ref: Rural)	1.00	–	–
	Urban	1.52	1.21–1.91	.003
Education	(ref: ≤Junior high)	1.00	–	–
	≥High school	0.92	0.69–1.22	.559
Widowed	(ref: No)	1.00	–	–
	Yes	0.94	0.68–1.31	.722
Self-rated health	(ref: Good)	1.00	–	–
	Poor	1.21	0.84–1.75	.306
Hypertension	(ref: No)	1.00	–	–
	Yes	1.33	0.95–1.86	.096
Hyperlipidemia	(ref: No)	1.00	–	–
	Yes	0.87	0.60–1.26	.457
Diabetes	(ref: No)	1.00	–	–
	Yes	1.28	0.89–1.84	.184
Stroke	(ref: No)	1.00	–	–
	Yes	3.01	2.18–4.16	<.001
Poor mood	(ref: No)	1.00	–	–
	Yes	3.22	2.02–5.14	<.001
Memory disorders	(ref: No)	1.00	–	–
	Yes	0.91	0.52–1.59	.739
Arthritis	(ref: No)	1.00	–	–
	Yes	1.12	0.79–1.59	.531
Smoking	(ref: No)	1.00	–	–
	Yes	2.14	1.58–2.90	<.001
Walk 1 km	(ref: Yes)	1.00	–	–
	No	0.89	0.66–1.20	.442
Depressive symptoms	(ref: No)	1.00	–	–
	Yes	1.13	0.85–1.50	.398

CI = confidence interval, OR = odds ratio.

### 3.4. Hierarchical logistic regression models for asthma risk

Table 4 details complete hierarchical regression results across 4 sequential adjustment stages. Protective effects for physical activity and air quality satisfaction remained significant throughout all adjustment stages. The final model confirmed persistent protective effects, with physical activity and air quality satisfaction maintaining significant risk reduction in the fully adjusted model (Table 4).

**Table 4:** Hierarchical logistic regression models for asthma risk (n = 3352).

Variable	Model 1	Model 2	Model 3	Model 4
	OR (95% CI)	OR (95% CI)	OR (95% CI)	OR (95% CI)
Physical activity				
High (Ref: low)	0.23 (0.14–0.38)	0.24 (0.15–0.40)	0.24 (0.15–0.41)	0.25 (0.15–0.41)
Air quality satisfied	0.31 (0.17–0.58)	0.32 (0.17–0.59)	0.32 (0.17–0.60)	0.33 (0.18–0.61)
Demographics				
Age (per 5-yr increase)	–	1.18 (1.05–1.33)	1.19 (1.06–1.35)	1.20 (1.06–1.36)
Urban (Ref: rural)	–	1.52 (1.21–1.91)	1.53 (1.22–1.92)	1.54 (1.23–1.94)
Female (Ref: male)	–	0.73 (0.48–1.11)	0.74 (0.49–1.12)	0.75 (0.49–1.14)
Health status				
Stroke	–	–	2.99 (2.17–4.12)	3.01 (2.18–4.16)
Hypertension	–	–	1.33 (0.95–1.86)	1.34 (0.96–1.88)
Arthritis	–	–	1.40 (0.98–2.01)	1.41 (0.98–2.03)
Lifestyle				
Current smoker	–	–	–	2.14 (1.58–2.90)
Poor mood	–	–	–	3.22 (2.02–5.14)
Model statistics				
Nagelkerke *R*^2^	0.13	0.19	0.25	0.29
Δ*R*^2^ from previous	–	+0.06	+0.06	+0.04
Hosmer–Lemeshow *P*	.42	.38	.41	.39

CI = confidence interval, OR = odds ratio.

### 3.5. Model performance and validation

The final hierarchical model explained 5.6% of asthma variance (Nagelkerke *R*^2^ = 0.056), with each adjustment stage providing significant incremental explanatory power (Δ*R*^2^ = 0.04 from Model 3 to 4, *P* < .001; Table 4). Sensitivity analysis confirmed the robustness of the main findings regarding PA, air quality satisfaction, and key risk factors (Table 5).

**Table 5:** Sensitivity analysis using backward stepwise regression for asthma risk factors.

Variable	Adjusted OR	95% CI	*P*-value
Physical activity			
High (≥23 MET-h/wk)	0.25	0.15–0.41	<.001
Air quality			
Satisfied	0.33	0.18–0.61	<.001
Urban residence	1.52	1.21–1.91	.003
Stroke history	2.99	2.17–4.12	<.001
Current smoking	2.14	1.58–2.90	<.001
Poor mood	3.22	2.02–5.14	<.001
Model fit statistics			
AIC	1827.5		
Nagelkerke *R*^2^	0.29		

AIC = Akaike information criterion, CI = confidence interval, MET = metabolic equivalent of energy, OR = odds ratio.

## 4. Discussion

This study provides robust evidence that modifiable lifestyle and environmental factors significantly impact asthma risk in China’s aging population. We demonstrate that maintaining higher physical activity levels (≥23 MET-h/wk) reduces asthma risk by 77%, while positive air quality perception decreases susceptibility by 69%. These findings clarify earlier ambiguities by establishing directional relationships: inadequate physical activity substantially increases asthma risk (OR = 3.85), and air quality dissatisfaction elevates risk by 222% (OR = 3.22). Crucially, these associations persist after comprehensive adjustment for demographic characteristics, health conditions, and lifestyle factors, underscoring their clinical relevance for preventive strategies targeting elderly populations with limited healthcare access.

The protective role of physical activity aligns with global evidence but offers novel quantification for Asian elderly.^[21]^ Our research findings go beyond studies focused on young people and demonstrate that appropriately customized exercise programs such as Tai Chi and brisk walking provide respiratory benefits in older adults without the risk of deterioration.^[22,23]^ These findings should help overcome therapeutic nihilism among healthcare providers regarding exercise prescription for elderly asthma patients.^[24]^ Notably, the 23 MET-h/wk threshold provides a concrete target for clinical recommendations, though implementation requires consideration of individual functional limitations and environmental barriers specific to China’s diverse regions.

Urban residents face 52% greater asthma risk than rural counterparts, a disparity explained through 3 evidence-supported pathways. Environmental monitoring studies have confirmed that the higher the PM content in urban centers, the more directly it triggers airway inflammation and increases the risk of death, as shown in hospitalization data from Shenyang.^[25,26]^ Immunological analyses reveal chronic urban stress dysregulates interleukin-6 pathways, increasing bronchial hyperreactivity.^[27,28]^ Furthermore, urban housing characteristics – smaller spaces, reduced ventilation, and synthetic materials – significantly increase indoor allergen exposure.^[29,30]^ These interconnected factors create cumulative biological burdens that disproportionately impact elderly urban residents with compromised respiratory reserves.^[31,32]^

The synergistic impact of comorbidities demands particular attention. Stroke history may significantly increase the risk of asthma by affecting the neuroinflammatory crosstalk in the respiratory control center, while psychological distress is more likely to increase susceptibility than traditional risks such as diabetes.^[33,34]^ This comorbidity triad – respiratory, cardiovascular, and mental health – necessitates integrated management protocols.^[35-37]^ Our results support 3 actionable strategies: policy interventions should prioritize particulate matter reduction in urban industrial zones; clinical guidelines must incorporate graded exercise prescriptions into standard asthma care; and research should investigate gene-environment interactions in high-risk subgroups using epigenetic approaches. Future longitudinal studies tracking inflammatory biomarkers could unravel the precise mechanisms linking these comorbidities.

## 5. Limitations

This study has several limitations. First, although we adjusted for key demographic, health, and lifestyle factors, residual confounding from unmeasured variables (e.g., environmental pollutant exposure levels, genetic susceptibility) may persist. Second, the cross-sectional design precludes causal inferences; longitudinal studies are needed to confirm the observed associations. Third, physical activity, asthma, and air quality satisfaction were self-reported, which may introduce recall bias. Future studies should incorporate accelerometer-based PA assessment and clinical asthma diagnosis.

## 6. Conclusions

This study demonstrates that higher physical activity levels and better air quality satisfaction are independently associated with significantly reduced asthma risk in elderly Chinese populations. These robust findings highlight the critical importance of integrating physical activity promotion and air quality improvement into public health strategies for asthma prevention. Policy makers should prioritize urban air pollution control interventions, while healthcare providers develop tailored exercise programs for elderly patients. Future longitudinal research should validate these associations and explore biological mechanisms, particularly focusing on gene-environment interactions in vulnerable subgroups.

## Acknowledgments

The authors thank all the participants in CHARLS team for their time and effort devoted to the project.

## Author contributions

**Conceptualization:** Caizhu Gao, Diyang Sun, Hang Yin, Yaqun Zhang, Yanan Pei.

**Data curation:** Caizhu Gao, Diyang Sun, Hang Yin, Yaqun Zhang, Yanan Pei.

**Formal analysis:** Caizhu Gao, Diyang Sun, Hang Yin, Yaqun Zhang.

**Funding acquisition:** Yanan Pei.

**Investigation:** Caizhu Gao, Diyang Sun, Hang Yin, Yaqun Zhang, Yanan Pei.

**Methodology:** Caizhu Gao, Diyang Sun, Hang Yin, Yaqun Zhang, Yanan Pei.

**Project administration:** Diyang Sun, Yanan Pei.

**Resources:** Hang Yin.

**Software:** Diyang Sun, Hang Yin.

**Writing – original draft:** Caizhu Gao, Yanan Pei.

**Writing – review & editing:** Caizhu Gao, Yanan Pei.
